# SNP‐RFLP Markers for the Study of *Arabidopsis lyrata*

**DOI:** 10.1002/ece3.71056

**Published:** 2025-04-23

**Authors:** Michelle Liu, Avery Chambers, Braidy Chambers, Alberto Aleman, Marc Stift, Katya Mamonova, Joanna Freeland, Marcel Dorken

**Affiliations:** ^1^ Department of Biology Trent University Peterborough Ontario Canada; ^2^ Environmental and Life Sciences Graduate Program Trent University Peterborough Ontario Canada; ^3^ Ecology, Department of Biology University of Konstanz Konstanz Germany; ^4^ Fraunhofer Institute for Molecular Biology and Applied Ecology IME Aachen Germany

**Keywords:** genetic resources for inbred populations, genome‐wide sequence data, mating systems, self‐compatibility, sequencing by transpose fragmentation, SNP discovery

## Abstract

*Arabidopsis lyrata* has become a useful system for the study of comparative genomics, hybridization, polyploidization, and evolutionary transitions from outcrossing to selfing. Previous studies of its mating system have used microsatellite loci, but low allelic diversity, particularly in self‐compatible populations characterized by low levels of outcrossing, reduces the utility of these markers for more detailed studies. Here, we aimed to develop population‐level SNP markers for *A. lyrata* ssp. *lyrata* sampled from a self‐compatible population at Rondeau Provincial Park, Ontario, Canada. We performed de novo SNP discovery and identified 6808 putative SNPs from genome‐wide sequences of 22 individuals originating from a highly selfing population. Further filtering and marker validation enabled the development of 17 SNP marker loci that can be visualized using standard PCR‐RFLP protocols. These markers had average minor‐allele frequencies of 0.40 in the target population, and four of seven markers were variable in a small sample from nine other *A. lyrata* populations. These PCR‐RFLP markers have the potential to be useful for the analysis of mating patterns within and beyond the inbred self‐compatible populations of *A. lyrata* studied here and enable the continued development of *A. lyrata* as a model for studying evolutionary transitions from outcrossing to selfing.

## Graphical abstract

We outline the development of highly variable, easy‐to‐use, and cost‐effective genetic markers for the analysis of *Arabidopsis lyrata* . These markers may be useful for the further development of this plant as a model for the study of micro‐evolutionary processes, including evolutionary modifications to plant mating systems.

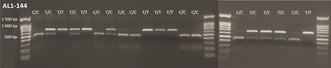

## 1 Introduction

The study of *Arabidopsis lyrata* has enabled progress across various areas of research. Its close relationship with *Arabidopsis thaliana* , combined with the publication of its genome (Hu et al. 2011), has encouraged studies of molecular evolution, including mutational load (Willi et al. 2018), transcriptional changes (Wos and Willi 2018), and transposons (Bonchev and Willi 2018). Population‐level research of *A. lyrata* has provided insights into evolutionary transitions between mating systems (Mable et al. 2005; Foxe et al. 2010), and the genomic causes (Li et al. 2023) and consequences of these shifts (Stift et al. 2013).

Studies of population‐level features for *A. lyrata* , including outcrossing rates and genetic diversity, have previously involved the analysis of microsatellite (short simple‐sequence repeat) markers (e.g., Foxe et al. 2010). Although microsatellites are relatively easy to use and can be highly polymorphic, they also have limitations to their utility, including problems with homoplasy and null alleles (Putman and Carbone 2014), low repeatability across labs (Miller et al. 2019), and their proneness to genotyping and scoring errors (Ball et al. 2010). An additional problem exists with the use of microsatellites for the study of *A. lyrata* —low levels of variation in self‐compatible and highly selfing populations (Mable and Adam 2007; Foxe et al. 2010). More advanced approaches to the study of genetic diversity, including the application of whole‐genome and genome‐wide sequencing to hundreds of individuals, have become more feasible (Ekblom and Wolf 2014; Christiansen et al. 2021), but might not be cost‐effective for studies that do not require whole‐genome data, such as the estimation of outcrossing rates from progeny arrays or investigations of population genetic structure.

Further progress in the study of population‐level processes would be enhanced by the development of single nucleotide polymorphism (SNP) based‐markers. SNPs have lower mutation rates, and their codominant and (mostly) biallelic nature can yield high‐quality genotype data from relatively simple genotyping assays (Morin et al. 2004). The development of multiple SNP marker loci can enable parentage analysis with similar resolving power as from more variable microsatellite loci (Flanagan and Jones 2019). For example, simulations and empirical studies have indicated that accurate parentage assignment is possible from as few as 20 loci, particularly when using SNP loci with high minor allele frequencies, either alone (Anderson and Garza 2006) or in combination with microsatellite loci (Labuschagne et al. 2015). Although genome‐wide SNP genotyping provides more data, simpler assays also avoid challenges associated with bioinformatic processing and biases caused by filtering decisions (Kratochwil et al. 2022).

Here, we outline the development and application of 17 easy‐to‐score single nucleotide polymorphism, restriction fragment length polymorphism (SNP‐RFLP) marker loci to complement an existing panel of nine microsatellite loci already developed for *A. lyrata* (Clauss et al. 2002; Mable and Adam 2007). We demonstrate that these SNP loci have high minor‐allele frequencies in a self‐compatible population of *A. lyrata* , are variable across populations, and are repeatable across labs.

## 2 Materials and Methods

### 2.1 Study Species and Population

*Arabidopsis lyrata* ssp. *lyrata* (L.) O'Kane & Al‐Shehbaz is a small, diploid perennial in the Brassicaceae that is—in most populations—the outcrossing relative of the highly selfing model plant *A. thaliana* (Mitchell‐Olds 2001; Mable and Adam 2007). The estimated size of its genome is 207 Mb, considerably larger than the 125 Mb genome of *A. thaliana* (Hu et al. 2011). Consistent with its history of outcrossing, there seems to be more genetic variation within the *A. lyrata* genome compared to the selfing *A. thaliana* (Johnston et al. 2005; Hu et al. 2011).

*Arabidopsis lyrata* is known to be mostly self‐incompatible (SI), but there have been at least two independent evolutionary transitions to self‐compatibility in North American populations (Foxe et al. 2010). Self‐compatible (SC) populations are characterized by high rates of self‐fertilization, but unlike other highly selfing relatives of SI plants, SC plants are morphologically similar to plants from SI populations (Carleial et al. 2017) and there is little evidence for inbreeding depression in SC populations (Li et al. 2019). Pollinators do not discriminate between plants from SC and SI populations, and there is no evidence of reproductive barriers between North American SI and SC populations upon secondary contact (Gorman et al. 2020).

High rates of inbreeding associated with low levels of microsatellite diversity in some populations of *A. lyrata* (Mable and Adam 2007; Foxe et al. 2010), and the problems associated with the use of microsatellites listed above motivated us to develop new, easy‐to‐use, and cost‐effective SNP‐RFLP markers. Because our ultimate aim was to conduct a detailed analysis of mating patterns in SC populations, we targeted a large SC population in Rondeau Provincial Park (RON) Ontario, Canada, for marker development. This population was previously identified as consisting entirely of SC individuals and is predominantly selfing (*t* = 0.28) (Mable et al. 2005; Mable and Adam 2007; Foxe et al. 2010). Analysis of microsatellite loci suggested no population subdivision, an inbreeding coefficient (*F*) of 0.41 (Mable et al. 2005), and low levels of expected heterozygosity (He = 0.03). Among self‐compatible North American populations, this population has been identified as one with high average density (Mable and Adam 2007).

### 2.2 DNA Extraction and Genome Sequencing

Seeds collected from 22 *A. lyrata* individuals located in Rondeau Provincial Park in Morpeth, ON, in July 2021 were germinated and placed in a climate‐controlled environmental chamber until the plants had produced at least 6 leaves. We harvested the above‐ground portion of these plants, dried them in coin envelopes placed in sealed bags containing silica beads. When the leaves were dry, they were ground to a semi‐fine powder using a Retsch MM300 mixer mill (Haan, Germany). DNA was extracted using a Fastpure plant DNA isolation mini kit (Nanjing Vazyme Biotech, China) following the manufacturer's protocol for dried material and eluted to a final volume of 100 μL using elution buffer. In total, DNA samples from 48 seedlings originating from 22 maternal plants (1–6 seeds per maternal plant) were extracted for subsequent sequencing. As described below, this larger sample was trimmed to include a single seedling per maternal parent to avoid introducing biases from the inclusion of related individuals from the same seed families in subsequent bioinformatic filtering steps.

DNA was quantified using a Qubit fluorometer (Thermo Fisher Scientific, USA) following the manufacturer's protocol. For each sample, the concentration was calculated as the average of three readings. Samples were either standardized to a concentration of 2 ng/μL or left undiluted if their concentration was below 2 ng/μL (0.4–2.0 ng/μL). To identify suitable SNP candidate regions, we obtained genome‐wide sequences from all 48 seedlings. The sequencing library was prepared using Illumina Tagment DNA enzyme (Illumina, USA) and buffer kit (small kit #20034210). Sequencing was done at The Centre for Applied Genomics (Toronto, Canada) using 126 bp Novaseq 6000 (Illumina, USA) and 51 bp Miseq (Illumina, USA.) paired‐end reads. All raw sequences are available on the Sequence Read Archive (SRA) (Leinonen et al. 2011) under the BioProject accession number PRJNA993789.

### 2.3 Variant Calling and SNP Discovery

Raw genome‐wide sequences of the 48 individuals were first checked for quality using FastQC v. 0.11.9 (Andrews 2019) and trimmed to remove adapters and low‐quality reads using the default Trimmomatic v. 0.39 (Bolger et al. 2014) settings, including the default LEADING:3 TRAILING:3 SLIDINGWINDOW:4:15 for trimming low‐quality reads. Only reads longer than 36 bp after trimming were retained. BWA‐mem v. 07.17 (Li 2013) was used to flag the positions of forward and reverse read fragments for each sample relative to a reference genome of *A. lyrata* ssp. *lyrata* strain MN47 (RefSeq assembly accession: GCF_000004255.2) obtained from the NCBI RefSeq database (O'Leary et al. 2016). We then used SAMtools v. 1.15.1 (Danecek et al. 2011) to sort and merge the aligned reads into one sequence alignment file representing the assembled genome‐wide reads of each seedling. To avoid biased estimates of MAF if some SNPs were over‐ or under‐represented in family groups, we used a trimmed sample size of 22 seedlings (one per maternal plant) for the variant calling and marker selection. When a maternal plant was represented by more than one offspring in the original sample group, we selected one representative offspring based on the best alignment score to the reference *A. lyrata* genome according to SAMtools *flagstat*.

### 2.4 In Silico Marker Development

To identify genetic variants, the assembled sequences of the 22 representatives were aligned to the reference genome using BCFtools mpileup (v. 1.9) (Danecek et al. 2021) using reads with minimum Phred read quality and mapping scores of 20. Variant calling was then done using BCFtools *call* with the multiallelic calling model, where locations with any sequence variation (including base variations, insertions, and deletions) between the samples and reference were identified and written to a Variant Call Format (VCF) file. This file stores information on variant positions, genotype likelihoods, as well as counts of reference alleles (identified from the *A. lyrata* reference genome) and alternative alleles as identified from the 22 samples. A total of 1,558,628 putative SNPs were identified after initial variant calling.

Our aims in filtering the data were to identify SNP markers that: (i) were biallelic, (ii) had a minor allele frequency (MAF) of at least 0.20, (iii) were not located in a duplicated region to avoid false SNP calls and spurious heterozygosity (Jaegle et al. 2023), and (iv) were not linked to other SNP markers developed here (i.e., the final set of markers were in linkage equilibrium).

The first stage of filtering was done using VCFtools v. 0.1.16 (Danecek et al. 2011) to remove insertion/deletion (INDEL) variants (–remove‐indels), fixed sites (–max‐non‐ref‐af 0.999) and any site where more than two alleles were called to retain only biallelic SNP sites (–min‐alleles 2 and –max‐alleles 2). We also used VCFtools to remove SNP sites where fewer than 15/22 samples were successfully genotyped (–max‐missing‐count 15) and relatively rare variants with MAF < 0.20 (–maf 0.20). These initial filtering steps left 6808 putative SNP sites for further development.

Significant deviations from Hardy–Weinberg Equilibrium (HWE) such as heterozygote deficiency can indicate population stratification or inbreeding, which we expected because of high selfing rates in our study population of *A. lyrata* (Foxe et al. 2010). Conversely, deviations from HWE in the form of heterozygote excess can indicate genotyping errors due to homologous regions in the *A. lyrata* genome (Wigginton et al. 2005; Jaegle et al. 2023). Short sequencing reads of duplicates may be mis‐mapped to only one region contained in the reference genome, resulting in “pseudo‐SNPs” or spurious excess heterozygosity (Jaegle et al. 2023). Thus, in order to avoid selecting potentially erroneous SNPs, we performed an exact test of HWE as defined by Wigginton et al. (2005) using VCFtools to detect SNP sites with significant HWE deviations. The *p*‐values for a lower‐tailed test of HWE to detect heterozygote deficit (pdeficit) and for an upper‐tailed test of HWE to detect heterozygote excess (pexcess) were also calculated using VCFtools to distinguish between the signals of inbreeding (heterozygote deficit) and of genotyping error (heterozygote excess). We then removed any SNP site with pexcess > 0.5, leaving 1123 putative SNPs.

These filtering steps yielded a list of candidate SNPs identified from a minimum of 15 samples. We further refined the list of SNPs by first selecting sites with 17 or more successfully genotyped samples. For sites with data from 16 plants or fewer, we selected only sites that displayed stronger evidence for heterozygote deficit (pdeficit < pexcess), as expected for a selfing population. Furthermore, for sites with data from only 15 samples, we performed a more stringent filtering for genotype quality by inspecting the sample read depth per site and only retaining sites where at least 11 samples were genotyped with a minimum read depth of 2. These additional filtering steps reduced the number of candidate SNPs to a total of 382. This total breaks down as follows: 82 candidate SNP loci had data from at least 17 samples, 142 candidate loci had data from at least 16 samples, and 158 candidate loci had data from at least 15 samples.

We wrote a Bash script utilizing SAMtools and BCFtools *consensus* to prepare FASTA files containing the flanking region (1000 bp up‐ and downstream) around each of the 382 SNPs identified as a possible genotyping target. We repeated variant calling using our original 48 samples and the reference *A. lyrata* genome to prepare another VCF file, which was then used for the preparation of the FASTA files to incorporate all possible base and length polymorphisms into the flanking sequences so that they could be taken into account in the subsequent restriction enzyme selection and primer design steps. Heterozygotes at polymorphic sites were marked with International Union of Pure and Applied Chemistry codes for the pair of alleles, while sites that failed to be genotyped were marked as “N.” Thus, each FASTA file represented one candidate SNP and contained the flanking sequences of all 48 seedlings as well as the reference *A. lyrata* genome. The Bash script mentioned here, along with other bioinformatic scripts, is available as indicated in the Open research statement.

To further reduce the incorporation of erroneous SNPs due to sequence duplications, we used the prepared FASTA files to run BLASTN (Altschul et al. 1990) local alignment search on a sequence of 41 bp (20 bp up‐ and downstream) and 201 bp (100 bp up‐ and downstream) around each of the 382 SNP sites against the reference *A. lyrata* genome, an additional *A. lyrata* ssp. *lyrata* genome assembly (GenBank assembly accession: GCA_944990045.1), as well as one *A. lyrata* ssp. *petraea* genome assembly (GenBank assembly accession: GCA_026151145.1). SNPs located within duplicate regions were removed from the candidate marker list, where we defined duplicates as homologous sequences that (i) occurred more than once within any of the three genomes included in the BLASTN search, (ii) overlapped with the SNP site, and (iii) were at least 90% identical to the query sequence.

### 2.5 Catalog and Genotyping Assay Development

We aimed to develop a simple SNP‐targeted assay with high repeatability and reproducibility. The SNP screening approach that we used in this study was PCR‐RFLP, using allele‐specific restriction enzyme (RE) digestion of PCR products (Jiang et al. 1996). With this method, primers were designed to flank a region with an RE recognition site that is present or absent depending on the SNP variant embedded within the recognition site. The region is amplified through PCR, and the resulting amplicons are digested using allele‐specific RE and then visualized with gel electrophoresis. Complete digestion of PCR products would appear as two bands smaller than the PCR amplicon, indicating that an individual is homozygous for the SNP allele recognized by the RE. Meanwhile, homozygotes of the alternate allele will result in no digestion (a single band the size of the expected PCR amplicon size). Heterozygotes are indicated by the presence of three bands on the gel due to digestion of one but not the other allele.

### 2.6 SNP Marker and Restriction Enzyme Selection

We visualized the aligned sequences contained in each FASTA file of the candidate SNP using the Multiple Sequence Alignment (MSA) Viewer (v. 1.23.0) available on the NCBI website (Kuznetsov and Bollin 2021), and examined the 2001 bp region (1000 bp up‐ and downstream of SNP site) for RE recognition sites using a publicly‐available tool by GenScript (“Restriction Enzyme Map Analysis” n.d.). Our aim was to generate RFLP markers based on each candidate SNP. Accordingly, the candidate SNP locus was embedded within the RE recognition site, such that samples homozygous for the allele corresponding with the RE recognition site would produce two bands in an agarose gel, samples homozygous for the alternative allele would produce a single (uncut) band, and heterozygotes would produce three bands (one uncut band and two bands from cut amplicons). We identified 49 candidate SNP markers embedded in a unique restriction enzyme recognition site with no duplicate recognition sites 150 bp up‐ or downstream of the SNP.

We used PLINK v 1.90 beta 6.21 (Purcell et al. 2007) to perform linkage analysis between the 49 candidate SNPs and to identify potentially linked markers (i.e., markers that should not be used in a multilocus analysis). The approximate degree of linkage was calculated using the Pearson's pairwise correlation coefficient (*r* ^2^) between the distribution of reference allele counts (0 for homozygotes of the alternative allele, 1 for heterozygotes, or 2 for homozygotes of the reference allele) for any two SNPs within a 1000 kbp window (Purcell et al. 2007). For every pairwise comparison that resulted in *r* ^2^ > 0.2, we considered the pair of SNPs to be in possible linkage disequilibrium and selected one of each putative linked pair to be included for the genotyping assay, favoring the SNP with less missing data (i.e., greater number of successfully genotyped samples), and by considering the accessibility of the associated restriction enzyme. In total, 20 putative SNP markers were chosen based on genotyping quality, accessibility of the restriction enzyme, and the absence of apparent linkage with other SNP marker sites. A summary of the SNP filtering and PCR‐RFLP marker selection pipeline is displayed in Figure S1.

### 2.7 Primer Design

For each SNP region, forward and reverse primers were designed using Primer‐BLAST (Ye et al. 2012) using default parameters including: GC% between 20% and 80%; primer melting temperatures 57°C–63°C; a maximum difference of 3°C melting temperature between primer pairs; and primer length between 15 and 25 bp. We aimed for PCR products to be at least 590 bp to ensure that digested fragments were large enough to visualize, and at most 1000 bp in length so that PCR products could be reliably produced.

In order to consistently amplify only the target region, primer design was limited to invariant sequences, and when possible avoided repetitive sequences. The flanking sequences (1000 bp up‐ and downstream of the SNP) were visually inspected on MSA Viewer to identify conserved regions among the 48 samples plus reference genome. Each primer was placed within a conserved region approximately 150–600 bp downstream or upstream of the SNP RE recognition site so that digested fragments would be at least 100 bp in size to enable clear visualization using agarose gel electrophoresis. For one target site (scaffold_60_7685_T_A) where this was not possible, we opted for primers that would amplify large (> 1300 bp) nontarget products (identified in silico) to enable separation between target and nontarget amplicons (n.b., this locus was later dropped during marker validation and optimization). To minimize noise on the gel electrophoresis, we further used the publicly available RE map analysis by GenScript (“Restriction Enzyme Map Analysis” n.d.) to check the sequences for duplicate recognition sites that might also be digested by the enzyme associated with the PCR‐RFLP primer pair.

### 2.8 Marker Validation

We used gradient PCRs to determine the optimal annealing temperature for each primer pair. For each marker locus, PCR reactions included 12.5 μL 2 × Froggabio (Concord, Canada) master mix, 0.5 μL of 10 mM forward and reverse primers, 1 μL DNA, and ddH_2_O for a final volume of 25 μL. Cycling conditions comprised an initial denaturation of 94°C for 2 min; 35 cycles of 45 s denaturation at 94°C, 45 s annealing (Table 1), 60 s extension at 68°C, and a final extension of 68°C for 2 min after the final cycle. We extended the number of cycles to 37 for locus 4 and 38 cycles for locus 9 (Table 1) to increase the amount of amplification product. PCR amplification was verified on a 1% agarose gel with a 100 bp ladder (Froggabio) for reference. We selected the temperature producing the brightest bands at the expected PCR product size when visualized in the gel. In addition to using gradient PCR, we amplified samples with varying starting DNA amounts extracted from a range of 20.0–0.2 mg dry material and eluted with 60 μL elution buffer from the Fastpure plant DNA isolation mini kit.

**TABLE 1:** SNP‐RFLP loci indicating forward (F) and reverse (R) primer sequences, primer annealing temperatures, amplicon fragment sizes (number of base pairs, bp), restriction enzymes for RFLP typing, the SNP allele at the RE recognition site, and fragment sizes (bp) up‐ and down‐stream of the SNP.

Locus	SNP ID^a^	Primer ID	Sequence (5′–3′)	*T* (°C)	Size	Enzyme	Digest allele	Size up	Size down
1	1_14411896_T_C	AL1‐144F	CGTTCAAAAGCGTGTCCTGTG	57.3	642	MnlI	C	200	442
		AL1‐144R	AGATTCCGTCTTGGATCATGGG						
2	1_26305924_A_G	AL1‐263F	GCTGTGATCTGCACGTTTTTG	58.5	807	BstCI	G	584	223
		AL1‐263R	TTGAGTTTGGACTGCCTTGGT						
3	1_28476668_A_G	AL1‐284F	ATGAGTTTGGTAAGCCTGACG	62.0	701	Hpy99I	G	187	718
		AL1‐284R	TTGTAAACCTTCGCAAAGCCC						
4	1_3626953_G_A	AL1‐362F	TCACCAATCACCATCGTTGAGA	62.5	768	AflIII	G	251	517
		AL1‐362R	TGTGTTATACTGGTTCCAAACTCT						
5	3_3650730_C_G	AL3‐365F	TGATGCGAGAAGTTGCTGTG	57.2	675	DdeI	C	234	441
		AL3‐365R	TCTGGCTCGTCAATTCCCTG						
6	4_16377278_G_T	AL4‐163F	CTGGCAGAGTTGTTCGACGG	64.2	752	NdeI	T	479	273
		AL4‐163R	GGTATGCTTCACTACACATCCG						
7	4_18833063_C_T	AL4‐188_2F	CGTATGCACCGCAAAAAGTACG	55.2	577	MseI	T	284	293
		AL4‐188_2R	TACGCGAAGACGCGGAAAC						
8	4_3731694_G_A	AL4‐373F	TGCTGAAACAAGGGAACTATCTGA	60.3	680	SmlI	A	451	229
		AL4‐373R	TCACTTCTTGCTGGTGCCAA						
9	5_16930178_G_A	AL5‐169F	CTGAGAGTGAGTCCACCAGT	59.0	860	Bsu36I	A	557	303
		AL5‐169R	TCCAAGTCAAATGGCTACCTC						
10	5_21025941_A_G	AL5‐210F	TGAGGTTTCTGGTTAGGATGGG	62.0	694	BclI‐HF	A	463	231
		AL5‐210R	CAGTTTGCAGTAACATGTGAAAACG						
11	6_7579614_C_T	AL6‐757F	CTCCCATGGGGCAGATGACT	51.0	789	NsiI‐HF	C	207	582
		AL6‐757R	AGCCTTGGTACTCATGAAAGTGT						
12	6_8478467_T_A	AL6‐847F	CACGAGCAAAGTGCGTGTTC	63.0	798	AseI	A	292	506
		AL6‐847R	GAGAGACGGTGGAGATACCG						
13	7_5932531_C_T	AL7‐593F	ACTGGTCGATATGCTGCTGT	59.5	579	MwoI	C	326	253
		AL7‐593R	GAGCTTTGTTCACCCTTGCG						
14	7_7632091_T_C	AL7‐763F	AGGCCGGAATACCTCCTAGC	55.4	646	NsiI	T	205	441
		AL7‐763R	CGTCCGGATCAGTTTCCCAT						
15	7_780813_C_T	AL7‐780F	ACGGTAAGCAATATTAGTTTTTCCA	54.2	650	SacII	C	406	244
		AL7‐780R	TGAAGGAAAGAGCAATGGGT						
16	8_14466046_C_T	AL8‐144F	GTTCTTCACAGCTTCTATGCTTCA	60.5	741	MspI	C	224	517
		AL8‐144R	GGAACTTCAGCTGCTAAGGA						
17	8_3829008_A_G	AL8‐382F	GAGGTGACTACAGGCCAAGA	58.0	806	CviQI	G	498	308
		AL8‐382R	CTGAAGGCCACTGGGGAATAGA						

^a^ SNP ID format: scaffold number_SNP position in bp_reference allele at SNP locus_alternative allele.

These PCRs were conducted using the samples with genotypes called from the sequencing data. We used a rotating subset of 4–6 previously sequenced samples to minimize depletion of DNA from individual samples in primer optimizations. Samples that were homozygous for the allele with the RE recognition site were included as putative positive controls. Alternative controls (negative digest controls) were also included in the validation tests using samples homozygous for the alternative allele. We also included samples identified from the sequence data as heterozygotes to demonstrate digestion of alternate PCR products. Negative controls with all PCR reagents and ddH_2_O instead of DNA were included for each optimization run.

**FIGURE 1:**
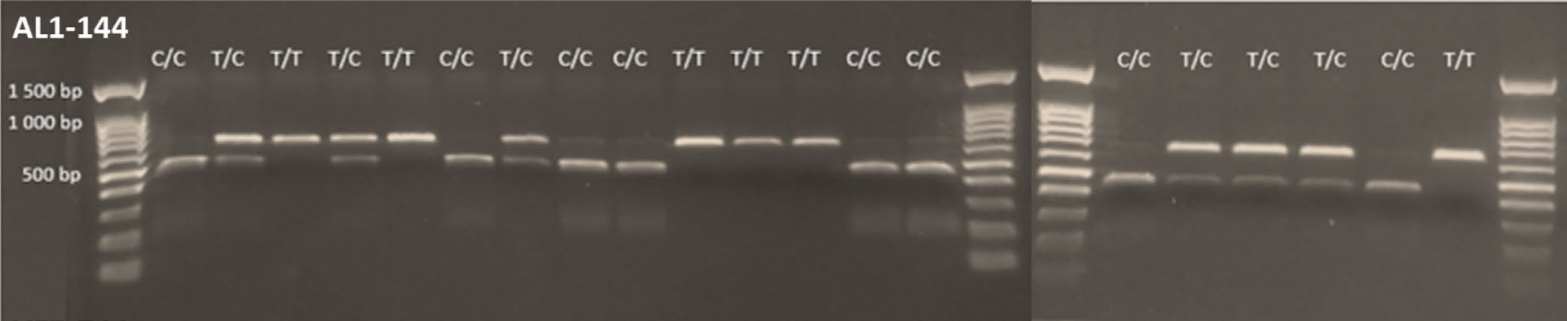
Example gel images depicting one of the PCR‐RFLP markers developed for *Arabidopsis lyrata* —locus 1, AL1‐144, digested using the MnlI restriction enzyme. For this locus, genotypes with the C allele at the SNP locus are digested by the restriction enzyme, yielding two fragments of 442 and 200 bp. Genotypes with the T allele produce an undigested amplification product of 642 bp. Heterozygotes can be identified by the presence of all three fragments. A complete set of example gel images for each locus is available in the Supporting Information.

Restriction enzyme digests were conducted using the associated enzyme for the target marker, using 4 μL of the amplified product with 1 μL RE buffer, between 0.25 and 0.75 μL of each enzyme (RE reactions conditions for each locus are provided in Table S1) and ddH_2_O for a total reaction volume of 10 μL. Samples were incubated at 37°C and visualized on a 1% agarose gel. A total of 17 loci produced repeatable and easily scored fragments of the expected sizes after RE digest (Figure 1). Different REs were needed for each locus, each corresponding to a different recognition site (Table 1). Some of the SNP loci involved the same pairs of alleles (e.g., 7 of 17 loci were polymorphic for A/G alleles), but they were not embedded in the same background sequence, meaning that each locus required a different RE.

After optimization, these 17 SNP‐RFLP markers underwent further testing by screening 20 seedlings from a new set of 20 maternal parents (one seedling per parent) from the RON population. For these plants, DNA extraction and amplification followed the same procedures as outlined above, with PCR and RE conditions as outlined in Table S1. To test the reproducibility of the markers in other labs and *A. lyrata* plants sourced from other North American populations, a random subset of seven marker loci was tested on two samples from each of 10 source populations (IND, LPT, MAN, PCR, PIN, PTP, RON, SBD, TSS, TSSA; codes and locations as in Foxe et al. 2010) at the Ecology lab at the University of Konstanz, Germany, using the same PCR and digest conditions as described above for the corresponding marker loci (i.e., without local optimization). These populations were also from the Laurentian Great Lakes region of North America and included a mix of populations comprised primarily of self‐compatible (4 populations) and self‐incompatible plants (6 populations).

## 3 Results

Sequencing yielded an average whole‐genome coverage across samples of 3.5×. We discovered 6808 putative biallelic SNPs by comparing the genome‐wide sequences of 22 individuals in the original sample. Exact tests for each SNP site indicated weak to moderate deviations from (0.05 < pHWE < 0.5) for 2745 sites, and significant deviations from HWE (pHWE < 0.05) for 745 sites. Lower‐tailed tests for HWE indicated some level of heterozygote deficiency (pdeficit < 0.5) for 373 sites. Upper‐tailed tests indicated increased heterozygosity (pexcess < 0.5) for 5685 sites, with 735 of those sites having significant heterozygote excess (pexcess < 0.05). This left 1123 remaining SNPs, 382 of which potential markers were deemed suitable for further testing based on genotyping quality.

Screening of the remaining sites based on genotyping quality, the presence of unique restriction enzyme recognition sites within the predefined sequence length of 2001 bp, linkage, and PCR repeatability yielded the 17 marker loci listed in Table 1. Most of these loci (9 of 17) had allele frequencies for the 20 additional samples from RON that were close to 50:50, and six out of the 17 markers were approximately 35:65 or below (Table 2). The most uneven allele frequency ratio was 17:83 (locus 8). Across all loci, the average minor allele frequency was 0.40 ± 0.10 SD (*n* = 20 per locus).

**TABLE 2:** Minor allele frequencies (MAF), the identity of the reference allele (RA) and the minor allele (MA) for each locus at the RON site, and from a sample of two plants from each of 10 additional populations tested at another lab for a random subset of the 17 loci developed here. Minor allele frequencies for RON were based on tests from the set of 20 plants used to optimize and test the markers, not the set of 22 plants used for genome sequencing. Reference alleles were based on the reference *Arabidopsis lyrata* genome; reference alleles and minor alleles had the same identity for some loci. The number of populations (including RON) observed to have multiple alleles is indicated in the final column.

Locus	MAF (RON)	RA (RON)	MA (RON)	MAF (10)	MA (10)	N polymorphic pop^b^
1	0.45	T	T	0.02	C	1
2	0.40	A	A	0.35	G	5
3	0.25	A	A			
4	0.47	G	A			
5	0.45	C	G			
6	0.50	G	G/T			
7	0.32	C	T			
8	0.17	G	G			
9	0.40	G	A	0.47	A	4
10	0.30	A	G			
11	0.35	C	T	0.02	C	1
12	0.47	T	A			
13	0.45	C	C			
14	0.32	T	T			
15	0.50	C	C/T	0.20	C	2
16	0.50	C	C/T			
17	0.45	A	G	0.15	G	1^a^

^a^ The two plants sampled from population PTP were homozygous for the minor allele.

^b^ Six of the seven loci tested at another lab provided results without further optimization.

Nontarget fragments were amplified for two marker loci (AL4‐188_2, AL4‐373). These two loci were not predicted to have nonspecific amplification in the primer design phase, but nontarget amplification occurred only in a few samples (Figure S2). Despite occasional nontarget PCR fragments, genotype identification remained clear because (1) only the PCR fragment of interest contained the RE site, and (2) nontarget PCR fragment sizes did not overlap with those of the target fragments.

Six of the seven loci tested in the Ecology lab, University of Konstanz, provided clear, scorable bands without any local optimization (Table 2). The seventh locus (locus 5) yielded RFLP gels that were too faint to score using unmodified protocols, and results from that locus are not included here. Even from a sample of only two plants per source population, half of the loci tested were polymorphic in other (self‐incompatible) populations of *A. lyrata* (MAN, SBD, and TSS; Table 2), with allele frequencies from the sample of additional populations similar to those at the RON site for two of the six loci.

## 4 Discussion

This study demonstrates the discovery and validation of 17 SNP‐RFLP loci based on the genome‐wide sequencing of 22 individuals originating from a highly selfing population of *A. lyrata* ssp. *lyrata* in Rondeau Provincial Park. The loci had high minor‐allele frequencies, making them potentially suitable for more detailed analyses, including the estimation of outcrossing rates and paternity exclusion. We demonstrated the repeatability of the markers across labs without further optimization, and even from a small sample, we were able to identify polymorphisms for some loci in other populations. SNP‐based PCR‐RFLP markers have been developed for other plants, including for the identification of species (e.g., in a hybrid zone; Chambers et al. 2024) or varietals of domesticated plants (Bazakos et al. 2012). In those other cases, many fewer loci were developed. We are not aware of the development of a similar‐sized battery of SNP‐RFLP markers for population‐level analysis.

From our filtered subset of 6808 putative SNP loci, we found that about 84% expressed some degree of increased heterozygosity compared to expectations under HWE. Generally, low heterozygosity is expected for highly inbreeding populations, and thus also for the highly selfing RON population studied here (Mable et al. 2005; Mable and Adam 2007; Foxe et al. 2010). The excess heterozygosity observed here might have reflected genotyping error—an issue commonly associated with low‐coverage sequencing data (Nielsen et al. 2011; Graffelman et al. 2017). Similar results were found for *A. thaliana* , for which 44% of SNPs called from 1135 accessions released by the 1001 Genomes Consortium were also found to be putatively heterozygous (Alonso‐Blanco et al. 2016), a large fraction of which were spurious and indicative of extensive duplications in the *A. thaliana* genome (Jaegle et al. 2023). We propose that the excess heterozygosity observed here, along with the amplification of nontarget regions for two of the loci developed in this study, reflects cryptic copy number variations in *A. lyrata* , though further studies into the genomic structure of highly heterozygous regions in *A. lyrata* would be needed to validate this. Moreover, pilot tests of these loci in previously uncharacterized populations are advised to more accurately ascertain allele frequencies.

To obtain a more representative population estimate of allele frequency distribution, we only used samples collected from different maternal plants, but this reduced the number of plants available for filtering based on minor allele frequencies (*n* = 12–19 per locus). Although as few as 25–30 individuals appear sufficient to estimate allele frequencies for a range of different taxa (Hale et al. 2012), our small sample size might have yielded loci with minor allele frequencies that missed our target of 0.20. Indeed, for one of our 17 loci, we detected a minor allele frequency of 0.17 in our sampling of 20 different plants from the RON population, and another locus with a minor allele frequency of 0.25. However, that still left us with 15 marker loci with minor allele frequencies of 0.30 or greater (Table 2), indicating that these loci, possibly in combination with already available microsatellite loci (Mable and Adam 2007) may be sufficient for parentage analysis (Anderson and Garza 2006; Labuschagne et al. 2015) and are more than sufficient for the estimation of outcrossing rates (Ritland 2002), or other detailed within‐population analyses of genetic variation (e.g., Vekemans and Hardy 2004).

Six out of seven loci were reproducible without optimization in an independent lab, and half of those were polymorphic based on small sample sizes from additional North American populations. These results suggest that the catalog of loci in Table 1 might be of broader utility for population‐genetic studies of North American *A. lyrata* . Additional testing of these markers is needed to confirm this; however, the three loci identified here as polymorphic across populations can be sufficient for applications such as the analysis of outcrossing rates (Ritland 2002). If further testing identifies additional loci that are polymorphic across populations, these could be used in parentage analyses from experimental arrays (e.g., Gorman et al. 2020; Steinecke et al. 2022), and broader investigations of population genetics such as the evolutionary relationships among populations (Kratochwil et al. 2022).

## Author Contributions

**Michelle Liu:** data curation (lead), investigation (equal), methodology (equal), writing – original draft (equal). **Avery Chambers:** data curation (equal), investigation (equal), validation (equal), visualization (equal), writing – review and editing (equal). **Braidy Chambers:** methodology (equal), validation (equal), visualization (equal), writing – review and editing (equal). **Alberto Aleman:** methodology (equal), resources (equal). **Marc Stift:** resources (equal), writing – review and editing (equal). **Katya Mamonova:** resources (equal), validation (equal), writing – review and editing (equal). **Joanna Freeland:** conceptualization (equal), funding acquisition (equal), supervision (equal), writing – review and editing (equal). **Marcel Dorken:** conceptualization (equal), funding acquisition (equal), project administration (lead), supervision (equal), writing – original draft (equal).

## Conflicts of Interest

The authors declare no conflicts of interest.

## Supporting information


**Figure S1.** Overview of the marker‐development pipeline.

**Figure S2.** Gel images for each of the 17 loci.

**Table S1.** PCR and RE conditions for each of the 17 loci.

## Data Availability

All data are available at the Sequence Read Archive under project number PRJNA993789. Data used to estimate allele frequencies at RON are provided in Figure S2. Additional notes on PCR and RE reaction conditions are provided in Table S1. Bioinformatic scripts are available at https://github.com/mchlleliu/AlyrataSNP_2023.
