# Supplementary material for: SNP‐RFLP Markers for the Study of Arabidopsis lyrata
Source: Ecol Evol. 2025 Apr 23;15(4):e71056. doi: 10.1002/ece3.71056 (PMC12015635; doi:10.1002/ece3.71056)

Supplementary figure S1. Variant calling and SNP filtering pipeline used to select candidate markers for paternity exclusion. A) Initial filtering processing of the raw VCF file to select SNP markers. B) HWE filtering to remove markers with signatures of duplicates and genotyping errors. C) Candidate marker selection and PCR-RFLP assay design.

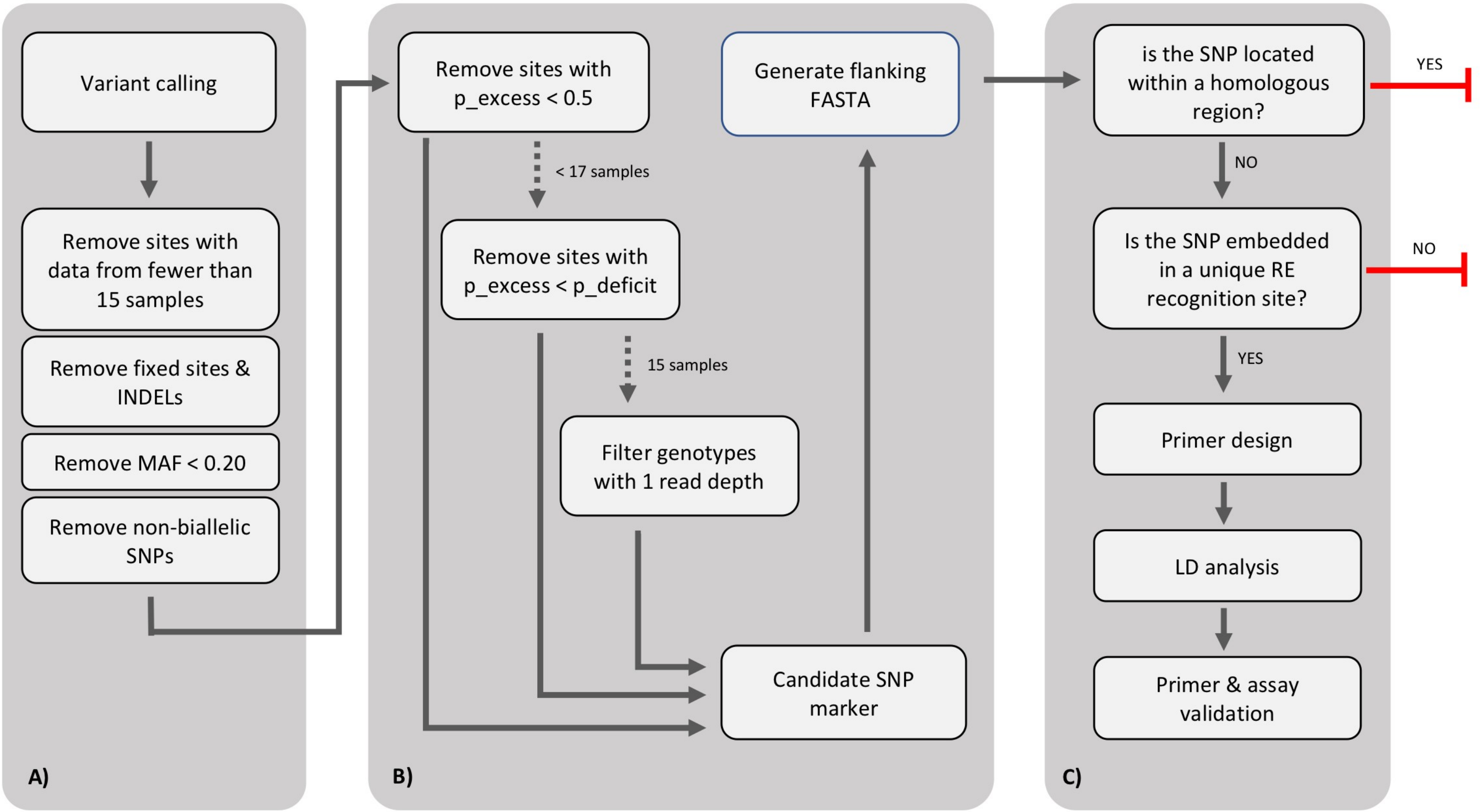

Supplement: Supplementary file 1 — Figure S1. Overview of the marker‐development pipeline. [file ECE3-15-e71056-s003.pdf]
