# Supplementary material for: SNP‐RFLP Markers for the Study of Arabidopsis lyrata
Source: Ecol Evol. 2025 Apr 23;15(4):e71056. doi: 10.1002/ece3.71056 (PMC12015635; doi:10.1002/ece3.71056)

Supplementary figure S2. Gel images for each locus showing the inferred PCR-RFLP genotypes for 12 *Arabidopsis lyrata* plants collected as seeds from different maternal parents in the self-compatible population at Rondeau Provincial Park, Ontario, Canada. Details for each locus, including the primer sequences and annealing temperatures can be found in Table 1 of the main document. Each locus image is flagged with the name of the primer set, encoded as AL for *A. lyrata*, the scaffold number, and the first three digits of the SNP position.

Locus 1

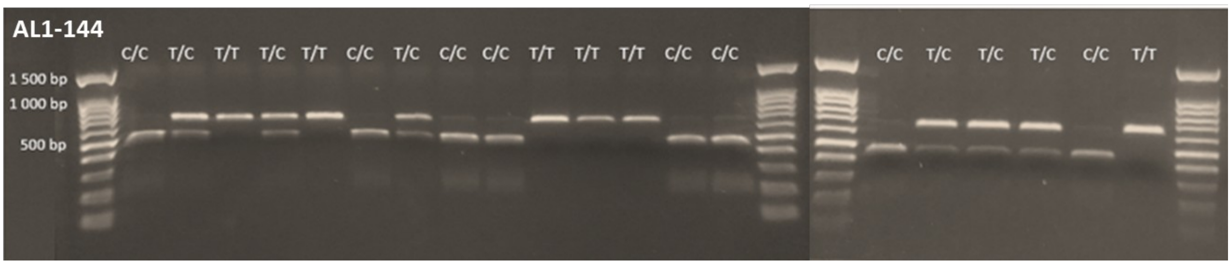


Locus 2

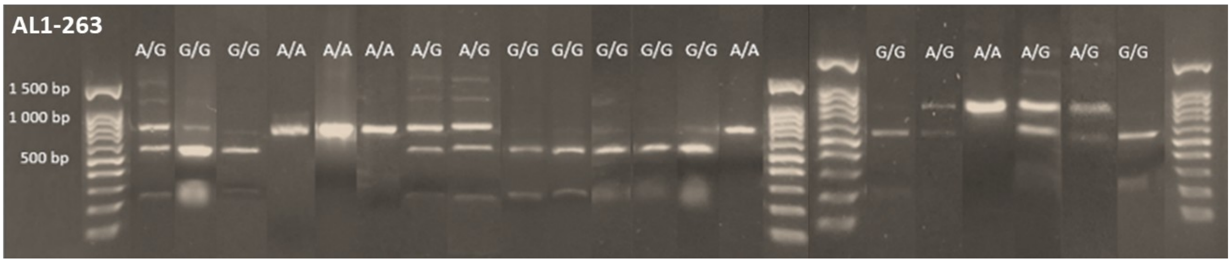


Locus 3

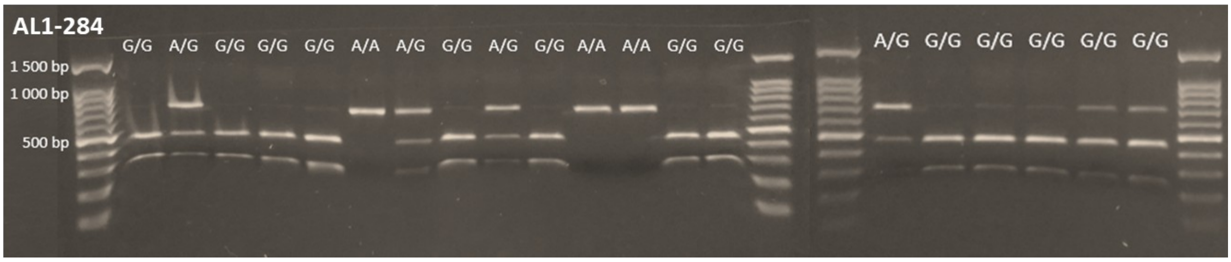


Locus 4

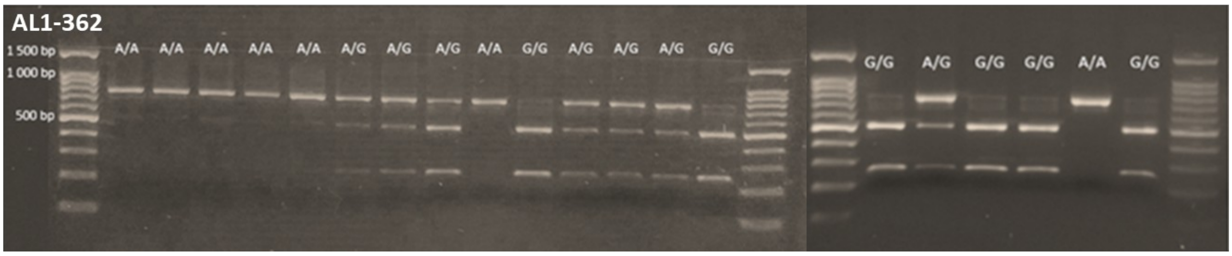

Locus 5

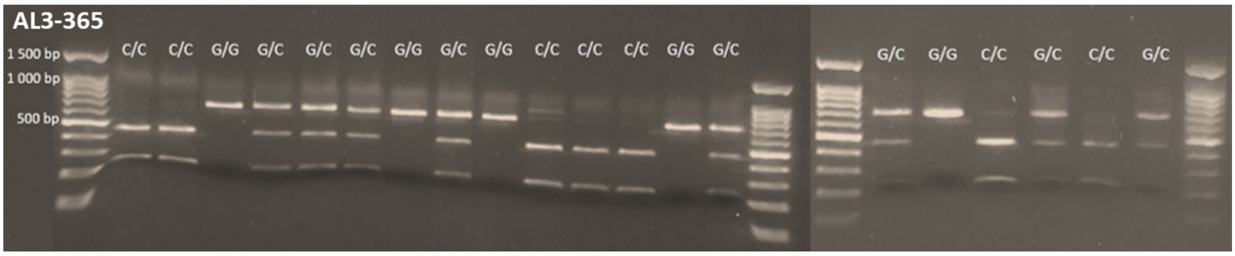


Locus 6

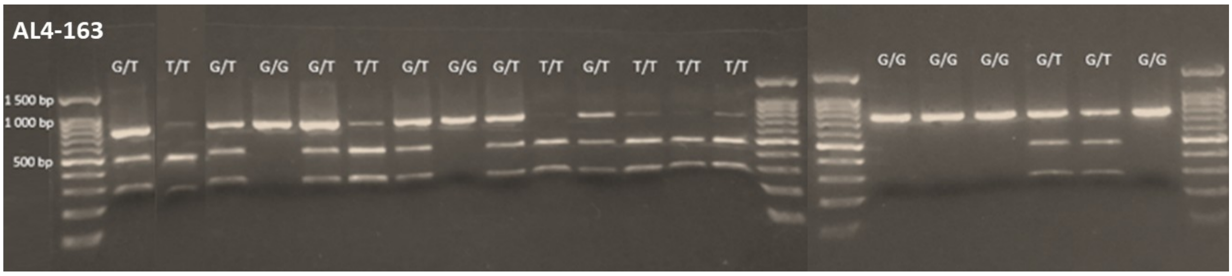


Locus 7

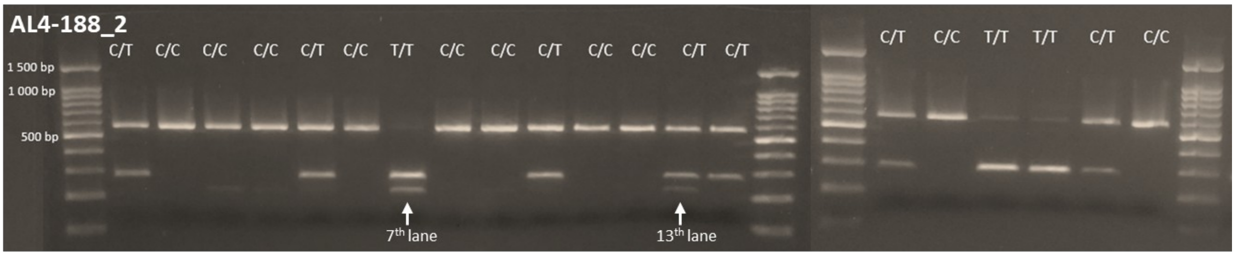


Locus 8

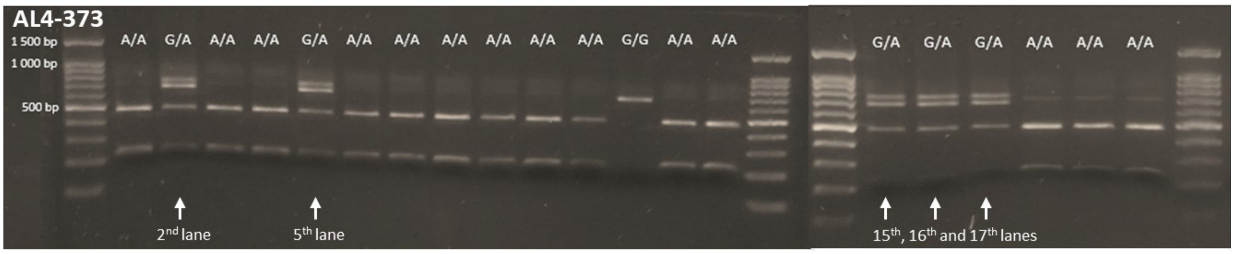

Locus 9

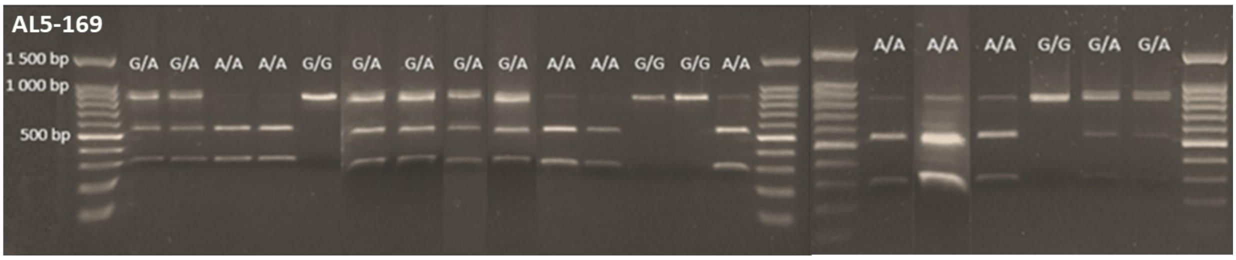


Locus 10

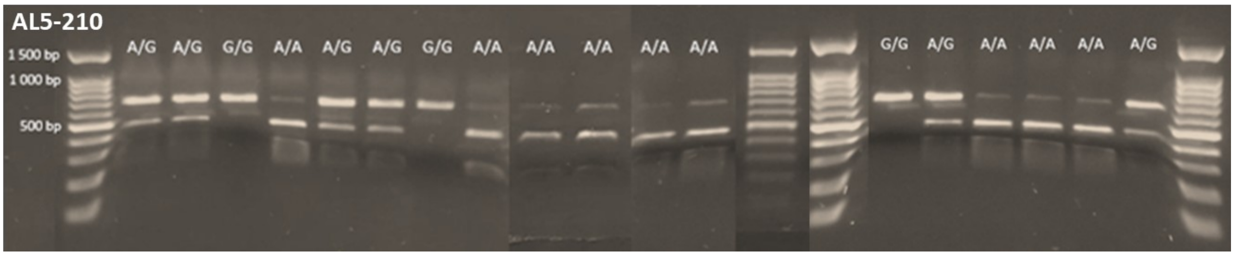


Locus 11

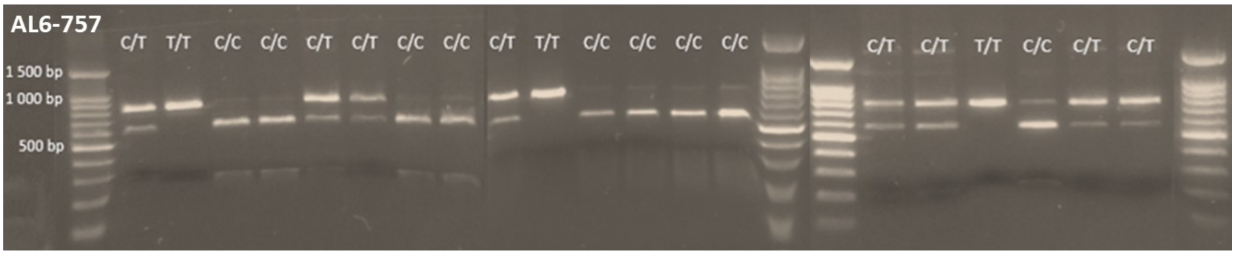


Locus 12

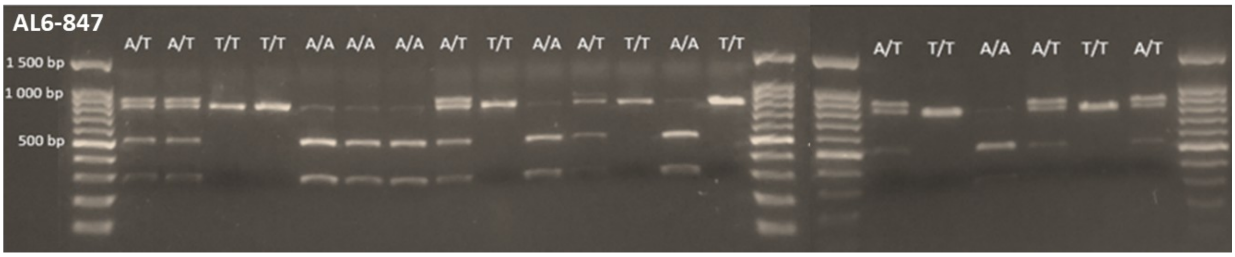


Locus 13

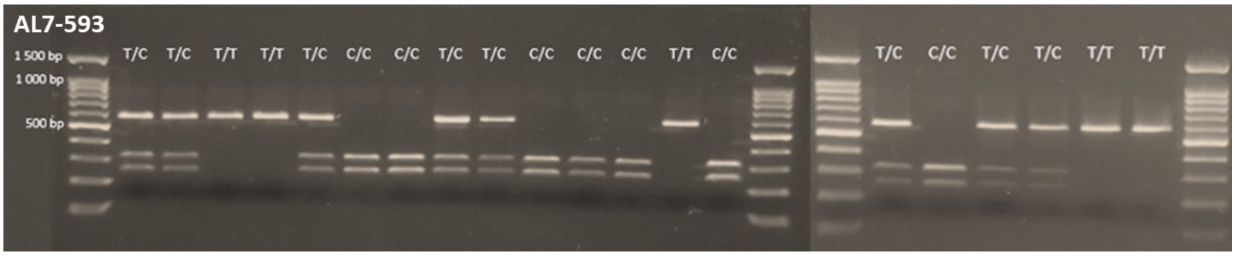

Locus 14

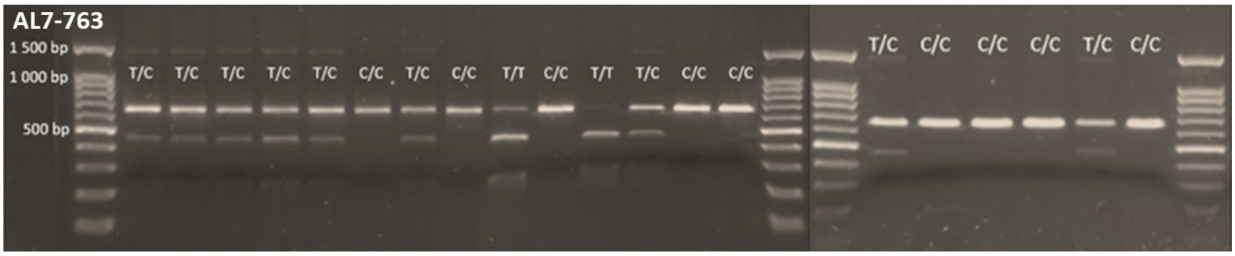


Locus 15

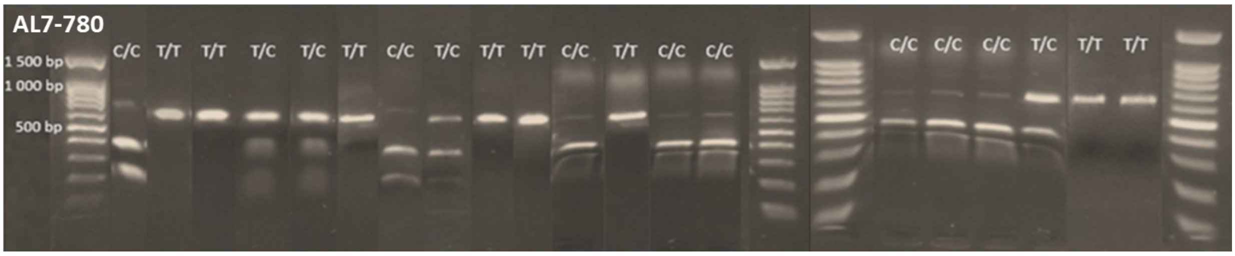


Locus 16

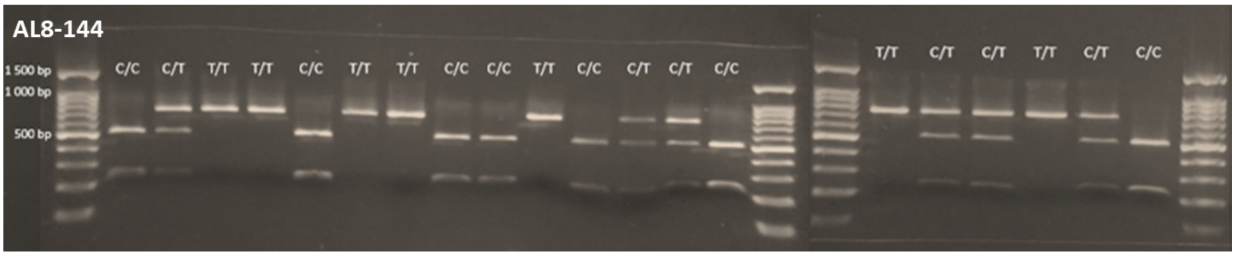


Locus 17

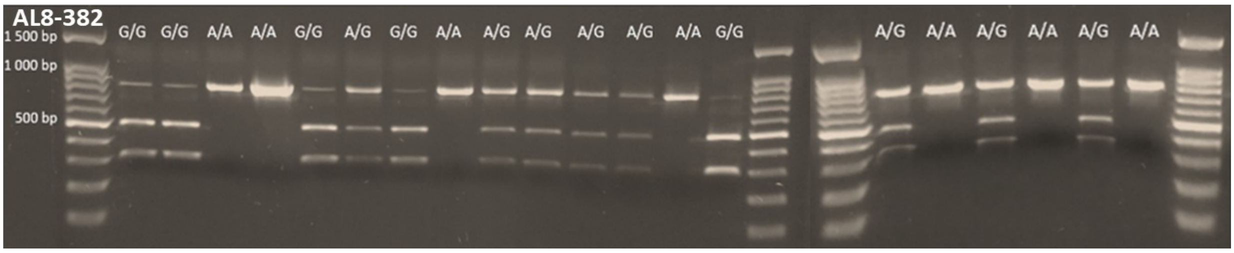

Supplement: Supplementary file 2 — Figure S2. Gel images for each of the 17 loci. [file ECE3-15-e71056-s001.pdf]
